# The role of Patronin in *Drosophila* mitosis

**DOI:** 10.1186/s12860-019-0189-0

**Published:** 2019-04-17

**Authors:** Gera A. Pavlova, Alyona V. Razuvaeva, Julia V. Popova, Evgeniya N. Andreyeva, Lyubov A. Yarinich, Mikhail O. Lebedev, Claudia Pellacani, Silvia Bonaccorsi, Maria Patrizia Somma, Maurizio Gatti, Alexey V. Pindyurin

**Affiliations:** 1 Institute of Molecular and Cellular Biology, Siberian Branch of the Russian Academy of Sciences, Novosibirsk, 630090 Russia; 2 Novosibirsk State University, Novosibirsk, 630090 Russia; 3 IBPM CNR and Department of Biology and Biotechnology, Sapienza University of Rome, 00185 Rome, Italy

**Keywords:** Patronin, CAMSAP proteins, Asp, Klp10A, Augmin, Dgt6, Mitosis, Microtubules, S2 cells, *Drosophila*

## Abstract

**Background:**

The calmodulin-regulated spectrin-associated proteins (CAMSAPs) belong to a conserved protein family, which includes members that bind the polymerizing mcrotubule (MT) minus ends and remain associated with the MT lattice formed by minus end polymerization. Only one of the three mammalian CAMSAPs, CAMSAP1, localizes to the mitotic spindle but its function is unclear. In *Drosophila*, there is only one CAMSAP, named Patronin. Previous work has shown that Patronin stabilizes the minus ends of non-mitotic MTs and is required for proper spindle elongation. However, the precise role of Patronin in mitotic spindle assembly is poorly understood.

**Results:**

Here we have explored the role of Patronin in *Drosophila* mitosis using S2 tissue culture cells as a model system. We show that Patronin associates with different types of MT bundles within the *Drosophila* mitotic spindle, and that it is required for their stability. Imaging of living cells expressing Patronin-GFP showed that Patronin displays a dynamic behavior. In prometaphase cells, Patronin accumulates on short segments of MT bundles located near the chromosomes. These Patronin “seeds” extend towards the cell poles and stop growing just before reaching the poles. Our data also suggest that Patronin localization is largely independent of proteins acting at the MT minus ends such as Asp and Klp10A.

**Conclusion:**

Our results suggest a working hypothesis about the mitotic role of Patronin. We propose that Patronin binds the minus ends within MT bundles, including those generated from the walls of preexisting MTs via the augmin-mediated pathway. This would help maintaining MT association within the mitotic bundles, thereby stabilizing the spindle structure. Our data also raise the intriguing possibility that the minus ends of bundled MTs can undergo a limited polymerization.

**Electronic supplementary material:**

The online version of this article (10.1186/s12860-019-0189-0) contains supplementary material, which is available to authorized users.

## Background

The spindle is the microtubule-based structure that mediates accurate chromosome segregation in both meiosis and mitosis. Microtubules (MTs) are highly dynamic and intrinsically asymmetric tubular structures formed by polymerization of α- and β-tubulin dimers [1]. In living cells, MTs mainly grow and shrink from their plus ends that expose β-tubulin. In vitro, MTs grow and shrink also from the minus ends where α-tubulin is exposed. However, in living cells the minus ends are more stable than the plus ends; they can depolymerize like the plus ends but are thought to have a very limited growing ability [1–3].

Many proteins have been identified that regulate MT plus end dynamics. They comprise several evolutionarily conserved families of MT plus-end tracking proteins that are collectively defined as +TIPs (microtubule plus ends tracking proteins). +TIPs include the End Binding proteins (e.g., EB1 and EB3 in vertebrates; DmEB1 in *Drosophila*) that accumulate at MT plus ends where they recruit other proteins such as the XMAP215/ch-TOG MT polymerase (Mini spindles, or Msps, in *Drosophila*), the CLASP proteins (MAST/Orbit/Chb in *Drosophila*) that regulate plus end polymerization, and MT depolymerases of the kinesin-13 family (e.g., mammalian MCAK and *Drosophila* Klp10A) and kinesin-8 family (human Kif18A and its *Drosophila* homologue Klp67A) [4–6]. However, it should be noted that while Klp10A behaves as a +TIP factor in interphase cells and induces MT plus end catastrophe, in mitosis it is primarily enriched at the spindle poles where it promotes MT depolymerization and poleward flux [7, 8].

Only a few MT minus end-associated proteins (−TIPs) have been so far described. The most characterized −TIP is γ-tubulin, which interacts with several additional subunits to form the γ-tubulin ring complexes (γ-TuRCs). γ-tubulin and its interacting partners in γ-TuRCs are evolutionarily conserved and act as templates for MT nucleation [9, 10]. γ-TuRCs mainly accumulate at centrosomes, promoting MT nucleation, but they also bind, protect and stabilize the free minus ends of preexisting MTs [11]. Another evolutionarily conserved multi-protein assembly that functions at the MT minus end is the octameric augmin complex (also called HAUS8 complex). The augmin complex contains two functional modules; one of these modules binds the walls of preexisting MTs, while the other module recruits γ-TuRCs, which nucleate new centrosome-independent MTs [12–14]. An additional minus end binding factor is the microcephaly-related ASPM protein, which has been recently shown to directly bind MT minus ends [15]. The localizations of ASPM and its *Drosophila* orthologue Asp appear to be restricted to the spindle, where they accumulate at the spindle poles and at the minus end-enriched extremities of the central spindles [16, 17]. Interestingly, +TIPs such as the EB proteins can also accumulate at polymerizing MT minus ends. This has been observed in vitro, but also in the few cases when MT minus end polymerization was detected in living cells [18].

Most of the +TIPs and −TIPs have been implicated in spindle assembly and functioning [4, 19]. Specifically, all +TIPs and −TIPs mentioned above are required for *Drosophila* spindle formation. For example, functional inhibition of *DmEB1, msps* or *Mast*/*Orbit*/*chb* results in short and morphologically abnormal spindles and impaired chromosome segregation [20–23]. In contrast, depletion of Klp10A, which preferentially depolymerizes MT minus ends, results in abnormally long spindles frequently showing a single aster nucleated by two collapsed centrosomes, but does not affect chromosome congression to the metaphase plate [23–25]. Klp67A depletion also results in long and often monastral spindles but impairs chromosome congression in metaphase [24–27]. Functional inactivation of genes encoding the somatic isoform of γ-tubulin (γ-tubulin 23C) or the other components of the γ-TuRCs (Dgrips, for *Drosophila* gamma ring proteins) causes similar mitotic phenotypes, including abnormally long, disorganized, monopolar and unpolarized spindles, often showing a reduced MT density [28–30]. A reduced MT density was also observed in augmin-depleted cells, which also show reduced kinetochore fiber (k-fiber) formation accompanied by defective chromosome alignment and segregation [12, 31]. Mutations in *asp* or *asp* RNAi cause abnormally long spindles with unfocused or split spindle poles as well as defects in chromosome congression, leading to a metaphase delay phenotype [16, 32, 33].

Recent studies have identified a new family of proteins that associate with the MT minus ends, the calmodulin-regulated spectrin-associated proteins (CAMSAPs/Patronin). In mammals, there are three CAMSAP proteins, CAMSAP1, CAMSAP2, and CAMSAP3/Nezha [34]. CAMSAP2 and CAMSAP3 bind and stabilize the minus ends of noncentrosomal MTs at the adherens junctions [35, 36]. Studies on interphase MTs and in vitro studies on isolated MTs have shown that CAMSAP2 and CAMSAP3 bind the slowly growing MT minus ends and remain bound to MTs when minus ends continue to polymerize, forming extended stretches of decorated MTs [37–39]. It has been further shown that CAMSAP2 and CAMSAP3 bind the MT severing ATPase katanin that disassembles the CAMSAP-decorated MT stretches, limiting their growth [38, 40]. CAMSAP2 and CAMSAP3 function in interphase but fail to associate with prometaphase and metaphase spindlesdue to phosphorylation [38, 41]. In contrast, CAMSAP1 localizes to the mitotic spindles but its loss only causes a small reduction in the spindle length [42].

There is only one *CAMSAP*-like gene in *Drosophila*, *Patronin*/*ssp4* (*CG33130*) that is most closely homologous to *CAMSAP3* [43, 44]. Patronin plays roles in both mitosis and the stabilization of non-mitotic MTs. Besides acting at the minus ends of interphase MTs, Patronin anchors and stabilizes the polarized noncentrosomal MT arrays of the *Drosophila* oocyte [45]. In mitosis, Patronin depletion leads to short spindles, a phenotype that is antagonized by Klp10A depletion, suggesting that Patronin caps the minus ends of the spindle MTs, preventing their depolymerization by Klp10A [43]. In addition, Patronin has been implicated in the control of anaphase B in *Drosophila* embryonic cells [46] and in the generation of central spindle asymmetry in asymmetrically dividing sensory organ precursor (SOP) cells [47]. However, there are still many aspects of the mitotic role of Patronin that need clarification. Specifically, Patronin localization during the mitotic phases and its precise mitotic role are poorly defined. The relationships between Patronin and other minus end associated factors such as Asp and augmin are also unexplored. Here, we address these issues and show that Patronin dynamically associates with different types of MT bundles within the *Drosophila* mitotic spindle, and that it is required for their stability. We also seek to define the functional relationships between Patronin, Klp10A, Asp and Dgt6 and propose a working hypothesis for the mitotic role of Patronin.

## Methods

### RNAi

Exon fragments of individual genes were amplified by PCR from genomic DNA isolated from wild-type (*Oregon-R*) flies. Primers used in PCRs contained at their 5′-ends the T7 RNA polymerase-binding site (5′-TAATACGACTCACTATAGGGAGG-3′), which was followed by a gene-specific sequence. The following gene-specific sequences were used: 5′-ATCGGACCATAA-3′ and 5′-TTGTTCTCGGCT-3′ for the *dgt6* gene [48], 5′-TTGCTGTCCATC-3′ and 5′-CGATCCTTGTCT-3′ for the *Klp10A* gene [49], 5′-CTGCGATCTTTCTTCAG-3′ and 5′-AGATGATTACGCCAATGC-3′ for the *asp* gene, and 5′-CGAGCTACAGCACCTGTTTC-3′ and 5′-TGACTGATTGCTGACATCGTCC-3′ for the *Patronin* gene. Synthesis of double-stranded RNAs (dsRNAs), as well as the subsequent procedure for RNAi in S2 cell cultures, were carried out as described previously [48]; dsRNAs were added to the cells twice (on the first and the third days of incubation) and cells were harvested for analyses after 5 days of RNAi.

The efficiency of RNAi was measured by reverse transcription followed by quantitative PCR (RT-qPCR) as reported previously [49], with the following modifications. Total RNA was isolated using RNAzol® RT reagent (MRC) according to the manufacturer’s instructions. Genomic DNA was eliminated using the RapidOut DNA Removal Kit (Thermo Fisher Scientific). Reverse transcription was performed with the RevertAid reverse transcriptase (Thermo Fisher Scientific) using 2 μg of total RNA in the presence of 2 U/μl of RNaseOut Recombinant RNase Inhibitor (Thermo Fisher Scientific). qPCR was carried out using the BioMaster HS-qPCR SYBR Blue (2×) reagent kit (Biolabmix; http://biolabmix.ru/en/). The following gene-specific primer pairs were used in qPCR: 5′-AACAGCTTACTCGCACCTGC-3′ and 5′-GCATGGGATCGTTGATCTTG-3′ for the *dgt6* gene, 5′-GCTGAGCGAACACGAGATGT-3′ and 5′-CAGTGTGGCATTAACGGTGC-3′ for the *Klp10A* gene, 5′-AAGTCGATTGGATCGTCTTTC-3′ and 5′-AATTTAGGATGATCCGGCTG-3′ for the *asp* gene, and 5′-TTTTCAAATACAACTCAGGAGGCA-3′ and 5′-ATTGTGAAGGCGTCGATGGT-3′ for the *Patronin* gene.

### Generation of stable S2 cell lines expressing fluorescently-tagged proteins

First, we designed a pair of primers (5′-ATGGATGTCGAAACACAGGAAATAC-3′ and 5′-GATTACAAGCGCCATGTCTTTTTTG-3′) to amplify full-length coding DNA sequences (CDSs) present in all 13 known transcript isoforms of the *Patronin* gene (FlyBase; [50]). These primers were used to obtain PCR products from cDNA made from total RNA isolated from S2 cells. The products were cloned into pGEM-T Easy plasmid vector (Promega), and several clones were partially sequenced. As a result, sequences of four different *Patronin* transcript isoforms were found, among which isoform I (encoding a polypeptide of 1689 amino acids) was present in almost half of the clones, suggesting that it could be the most abundant transcript isoform of this gene in S2 cells. The cloned CDS of isoform I was fully sequenced and the following variations were identified in the encoded amino acid sequence relative to the expected one (GenBank accession no. NP_001261051.1): Q743del, T859A and V1481I. The last two variants are also found in the previously reported amino acid sequence of Patronin (GenBank accession no. AFA36631.1). As an alternative, we PCR-amplified *Patronin* CDS isoform A (encoding a polypeptide of 1517 amino acids) using as a template genomic DNA isolated from transgenic flies expressing GFP-tagged version of this isoform (w[*]; *P{w[+mC] = Ubi-p63E-Patronin.A.GFP}3 M/TM3, Sb*; Bloomington stock no. 55129). *Patronin* CDS isoform A was also cloned and sequence verified; it encodes the protein with the expected amino acid sequence (GenBank accession no. NP_788398.1).

The only known full-length CDS isoform of the *asp* gene (FlyBase; [50]) was PCR-amplified using cDNA made from total RNA isolated from S2 cells as a template and the appropriate primers (5′-ATGAGCGCCTTTGAGATCACAGTGA-3′ and 5′-AAACATGTCGATCTGCAGCTTGCAC-3′). It was subsequently cloned and verified by sequencing, which revealed the following two variations in the encoded amino acid sequence relative to the expected one (GenBank accession no. NP_524488.3): W196R and G1662D.

These three sequence-verified full-length CDSs were cloned in a piggyBac transposon-based plasmid vector upstream of, and in-frame with, a DNA sequence encoding enhanced GFP (EGFP; hereafter, for simplicity, referred to as GFP). The plasmids also contained a blasticidin-resistance cassette and the sequence encoding mCherry-α-tubulin (hereafter Cherry-tubulin) fluorescent fusion protein. The expression of all fluorescent fusion proteins was controlled by the copper-inducible *Metallothionein* *A* (*MtnA*) promoter. S2 cells co-transfected with a plasmid encoding fluorescently-tagged fusion proteins and a plasmid encoding piggyBac transposase were cultured in medium supplemented with 20 μg/ml blasticidin (Sigma) for 2 weeks at 25 °C. The antibiotic was then removed from the culture medium. All cells were free from mycoplasma contamination. To induce expression of fluorescent fusion proteins, cells were grown in the presence of 250–500 μM copper sulfate for 12–17 h before in vivo analysis or fixation.

### Cytological procedures

All procedures were performed at room temperature. For cytological analysis of mitosis, 2 × 10^6^ S2 cells were centrifuged at 800 *g* for 5 min, washed in 2 ml of PBS (Sigma), and fixed for 10 min in 2 ml of 3.7% formaldehyde in PBS. Fixed cells were spun down by centrifugation (at 800 *g* for 5 min), resuspended in 500 μl of PBS and placed onto a clean slide using a Cytospin™ 4 cytocentrifuge (Thermo Fisher Scientific) at 900 rpm for 4 min. The slides were immersed in liquid nitrogen, washed in PBS, incubated in PBT (PBS with 0.1% TritonX-100) for 30 min and then in PBS containing 3% BSA for 30 min. The slides were then immunostained using the following primary antibodies, all diluted in PBT: mouse anti-α-tubulin (1:600, Sigma T6199), rabbit anti-Spd2 (1:1000, [51]) and chicken anti-GFP (1:200, Invitrogen PA1-9533). These primary antibodies were detected by incubation for 1 h with goat FITC-conjugated anti-mouse IgG (1:40, Sigma F8264) or goat Alexa Fluor 568-conjugated anti-mouse IgG (1:300, Invitrogen A-11031), goat Alexa Fluor 568-conjugated anti-rabbit IgG (1:300, Invitrogen A-11036) or goat Alexa Fluor 660-conjugated anti-rabbit IgG (1:300, Invitrogen A-21074), and goat Alexa Fluor 488-conjugated anti-chicken IgG (1:300, Invitrogen A-11039). Slides were mounted in Vectashield with 4,6-diamidino-2-phenylindole (DAPI) (Vector Laboratories) or in ProLong™ Gold Antifade Mountant with DAPI (Thermo Fisher Scientific) to stain DNA and reduce fluorescence fading. Images of fixed cells were captured by an AxioCam 506 mono (D) camera (Carl Zeiss) using a ZeissAxioImager.M2 with an EC Plan-Neofluar 100×/1.30 oil lens (Carl Zeiss).

### Live cell imaging

500 μl aliquots of suspended cells (1 × 10^6^ cells/ml) that express fluorescently-tagged proteins were transferred to cell chambers (Invitrogen A-7816) containing coverslips treated with 0.25 mg/ml concanavalin A (Sigma-Aldrich C0412). Observations were made between 20 and 120 min after cell plating. Images of living cells were obtained on a Zeiss LSM 710 confocal microscope using a plan-apo 63×/1.40 oil lens and the ZEN 2012 software.

## Results

### Patronin associates with spindle MT bundles

The *Patronin* gene produces at least 13 distinct protein isoforms, all showing different amino acid sequences (see FlyBase, [50]). To address the mitotic role of Patronin, we focused on the isoforms I and A that contain 1689 and 1517 amino acids, respectively. We chose to focus on isoform I because it is likely to be one of the most abundant Patronin isoforms in S2 cells (see Methods), while isoform A was used in the study due to the availability of transgenic flies expressing it as a GFP-tagged fusion protein. We generated two S2 cell lines expressing Cherry-tubulin and either Patronin isoform I-GFP or Patronin isoform A-GFP, all under the control of a copper-inducible promoter. After induction of the transgenes (see Methods), cells were fixed and stained with anti-GFP and anti-α-tubulin antibodies. An analysis of mitotic preparations revealed no difference in the staining pattern of the two Patronin isoforms. Thus, in all experiments described below we used the cell line expressing Patronin isoform I-GFP, unless otherwise specified.

**Fig. 1:**
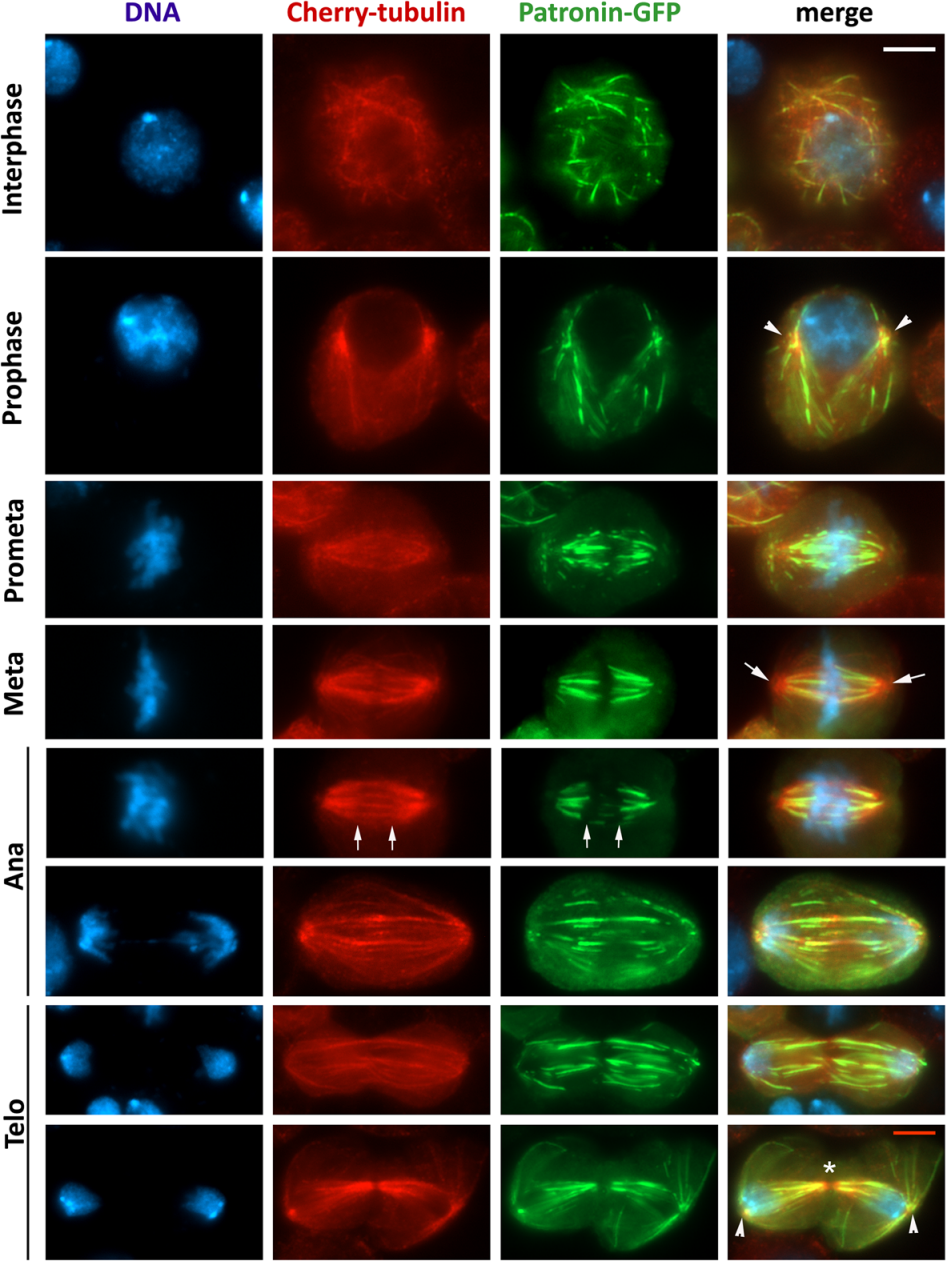
Patronin-GFP localizes to a subset of the MT bundles that compose the mitotic spindle of S2 cells. Cells expressing Patronin-GFP and Cherry-tubulin were fixed and stained with anti-GFP (green) and anti-α-tubulin (red) antibodies, and with DAPI to detect DNA (blue). Prometa, prometaphase; Meta, metaphase; Ana, anaphase; Telo, telophase. Note that only a subset of the MT bundles associate with Patronin-GFP. In prometaphase, metaphase and early anaphase cells, the extremities of the Patronin-stained bundles are mostly excluded from the spindles poles (arrows in the metaphase cell), whereas in prophase and late telophase figures some bundles end at the center of the asters (arrowheads). Notably, in early anaphase cells the thin MTs bundles at the center of the cell (delimited by two arrows) are not enriched in Patronin. In late anaphase and telophase cells, the middle region of the central spindle, where the plus ends of antiparallel MTs overlap, is not associated with Patronin (asterisk). The white scale bar (5 μm) refers to all cells except telophases (red scale bar, 4 μm)

Interphase cells showed stretches of Patronin-GFP-associated MT bundles of variable length. Some cells showed only short segments of GFP-stained MT bundles that are presumably enriched in minus ends, while other cells displayed long MT bundles decorated by Patronin (Fig. 1). In prophase cells, Patronin associates with some but not all the MT bundles emanating from the centrosomes, and the staining patterns displayed by different cells were rather variable in terms of the number of “astral bundles” decorated by Patronin (Fig. 1; Additional file 1: Figure S1). Prometaphase cells were even more variable than prophases, as they showed different numbers of Patronin-decorated bundles within the spindle. In addition, the Patronin positive stretches in a bundle were quite variable in length, and some bundles showed two or more Patronin-associated segments separated by Patronin-negative regions (Fig. 1; Additional file 1: Figure S1). In metaphase cells, most k-fibers appeared to be continuously, or almost continuously, decorated by Patronin. However, some long MT bundles (presumably interpolar bundles) were not associated with Patronin (Fig. 1; Additional file 1: Figure S1). In early anaphase cells, all k-fibers were decorated by Patronin-GFP, while the thin MT bundles at the center of the cell (probably bridging fibers, [52]) were usually not stained or weakly stained by anti-GFP antibodies (Fig. 1). In late anaphases and telophases, most tubulin bundles were partially or totally decorated by Patronin-GFP. However, the middle region of the central spindle, where the MT plus ends overlap, was invariably devoid of Patronin (Fig. 1; Additional file 1: Figure S1).

**Fig. 2:**
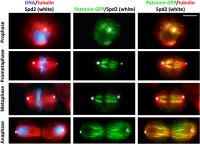
In prometaphase and metaphase cells Patronin-enriched MT bundles are separated from the centrosomes. Cells expressing Patronin-GFP and Cherry-tubulin were fixed and stained with chicken anti-GFP, mouse anti-α-tubulin and rabbit anti-Spd2 antibodies, which were detected using secondary antibodies conjugated with Alexa Fluor 488 (green), Alexa Fluor 568 (red) and Alexa Fluor 660 (far red; white), respectively. Note that in prometaphase and metaphase cells the fluorescent MT bundles do not reach the centrosomes, whereas in prophase and telophase cells at least some bundles contact the centrosomes. Scale bar, 5 μm

To obtain insight into the relationship between Patronin and centrosomes, we determined the subcellular localization of Patronin relative to the centrosomal marker Spd2 [51]. The analysis of cells expressing Patronin-GFP stained with anti-GFP, anti-Spd2 and anti-α-tubulin antibodies revealed that in prophase cells Patronin-stained MT bundles depart from centrosomes or from near the centrosomes (Fig. 2). In prometaphase, metaphase and early anaphase, the centrosomes were invariably separated from the Patronin-positive MT bundles by an area stained only by the anti-α-tubulin antibodies (Fig. 2). In telophase cells, the Patronin stained “astral bundles” departed from the centrosomes or from near the centrosomes like in prophase (Fig. 2). These findings suggest that, with the possible exception of prophase and telophase, Patronin does not bind the MT minus ends embedded in the centrosomes. We note that Patronin localization during mitosis is reminiscent of the localization of the Dgt6 augmin subunit [31]. Although in prophase and telophase cells Dgt6 is enriched at the center of the asters, in prometaphases and metaphases it does not accumulate in these spindle regions [31], just as Patronin. In addition, both Patronin and Dgt6 fail to localize to the middle region of the telophase central spindle ([31], Figs. 1, 2; Additional file 1: Figure S1).

### Patronin behavior in mitotic spindles is dynamic

**Fig. 3:**
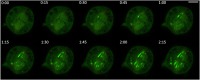
Patronin exhibits dynamic behavior during prometaphase in S2 cells. Stills from a time-lapse video of an S2 cell prometaphase expressing Patronin-GFP. The numbers at the top of each frame indicate the time (min:sec) elapsed from the beginning of imaging. Note that Patronin-GFP seeds appear near the chromosomes (non-fluorescent dark spots) and extend towards the spindle poles along preexisting MT bundles, revealing an unexpected dynamic behavior of Patronin. See Additional file 3: Movie S1. Scale bar, 5 μm

**Fig. 4:**
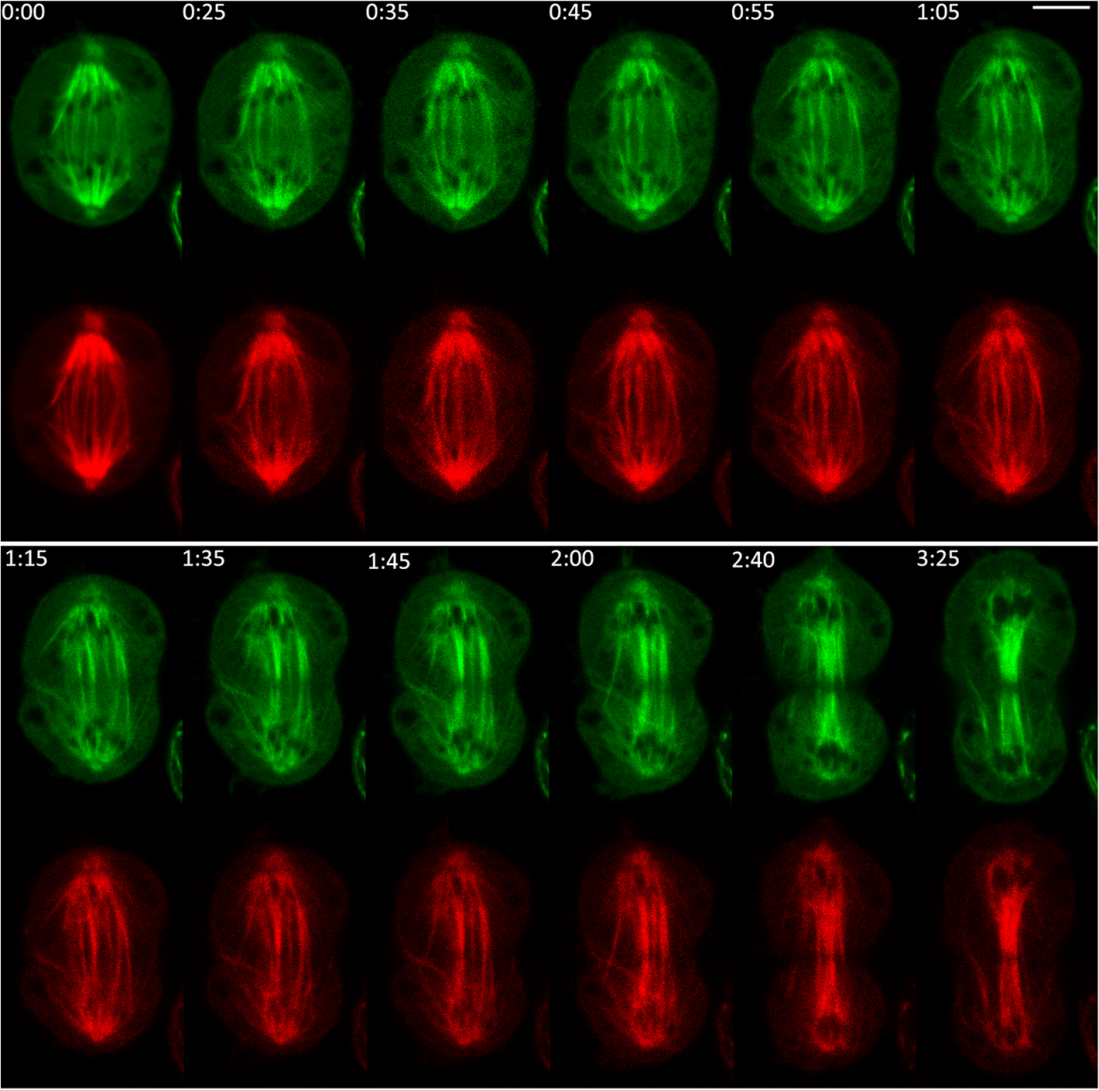
Patronin-GFP behavior during ana-telophase of S2 cells. Stills from a time-lapse video of an S2 cell expressing Patronin isoform A-GFP (green) and Cherry-tubulin (red), followed from anaphase to telophase. The numbers at the top of each frame indicate the time (min:sec) elapsed from the beginning of imaging. Note that the chromosomes appear as dark spots in the green Patronin isoform A-GFP background. See Additional file 5: Movie S2. Scale bar, 5 μm


The variability of the Patronin staining pattern in mitotic spindles suggests that Patronin can move along the spindle MT bundles and that the microphotographs of formaldehyde-fixed cells are capturing the instantaneous positioning of this protein along the spindle. To address this issue, we imaged living cells expressing Patronin-GFP, focusing on prometaphase cells that show the most variable Patronin staining pattern in fixed material, and we found that Patronin displays a highly dynamic behavior. In some prometaphase cells, we saw Patronin accumulating on short segments of MT bundles located near the chromosomes. These short, brightly fluorescent regions extended towards the cell poles and stopped growing just before reaching the poles (Fig. 3; Additional file 2: Figure S2; Additional file 3: Movie S1). This behavior is consistent with our observations of fixed material indicating that in prometaphase cells Patronin-positive MT bundles never reach the spindle poles and are always separated from the centrosomes (compare Fig. 3; Additional file 2: Figure S2 with Fig. 2). We calculated the velocity at which the fronts of Patronin-fluorescent seeds extend along the MT bundles and found that they move at a velocity of 1.44 ± 0.16 μm/min. We also examined the behavior of Patronin during metaphase, early and late anaphase, and telophase. In metaphase and early anaphase spindles, Patronin is much less dynamic than in prometaphase spindles. In late anaphase and early telophase cells, Patronin mostly followed the dynamic behavior of tubulin. In ana-telophase cells we did not see Patronin extending along MT bundles from highly fluorescent seeds as in prometaphase cells. Instead, while the spindle poles were separating during anaphase B, Patronin-enriched MT bundles seemed to emanate from the stubs of the kinetochore MTs located between the centrosome and the chromosome set. These bundles extended towards the center of the cells while recruiting additional Patronin, and eventually gave rise to the telophase central spindle (Fig. 4; Additional file 4: Figure S3; Additional file 5: Movie S2). The Patronin stretches associated with the “astral” MT bundles also appeared to extend away from centrosomes toward the cell periphery (Fig. 4; Additional file 4: Figure S3; Additional file 5: Movie S2).

**Additional file 3:** **Movie S1.** A time-lapse video of an S2 cell prometaphase expressing Patronin-GFP (green). The numbers at the top indicate the time (min:sec) elapsed from the beginning of imaging. Scale bar, 5 μm. (MOV 278 kb)



**Additional file 5:** **Movie S2.** A time-lapse video of an S2 cell expressing Patronin isoform A-GFP (green) and Cherry-tubulin (red), followed from anaphase until telophase. The numbers at the top indicate the time (min:sec) elapsed from the beginning of imaging. Scale bar, 5 μm. (MOV 442 kb)


### Functional relationships between Patronin and Klp10A

**Fig. 5:**
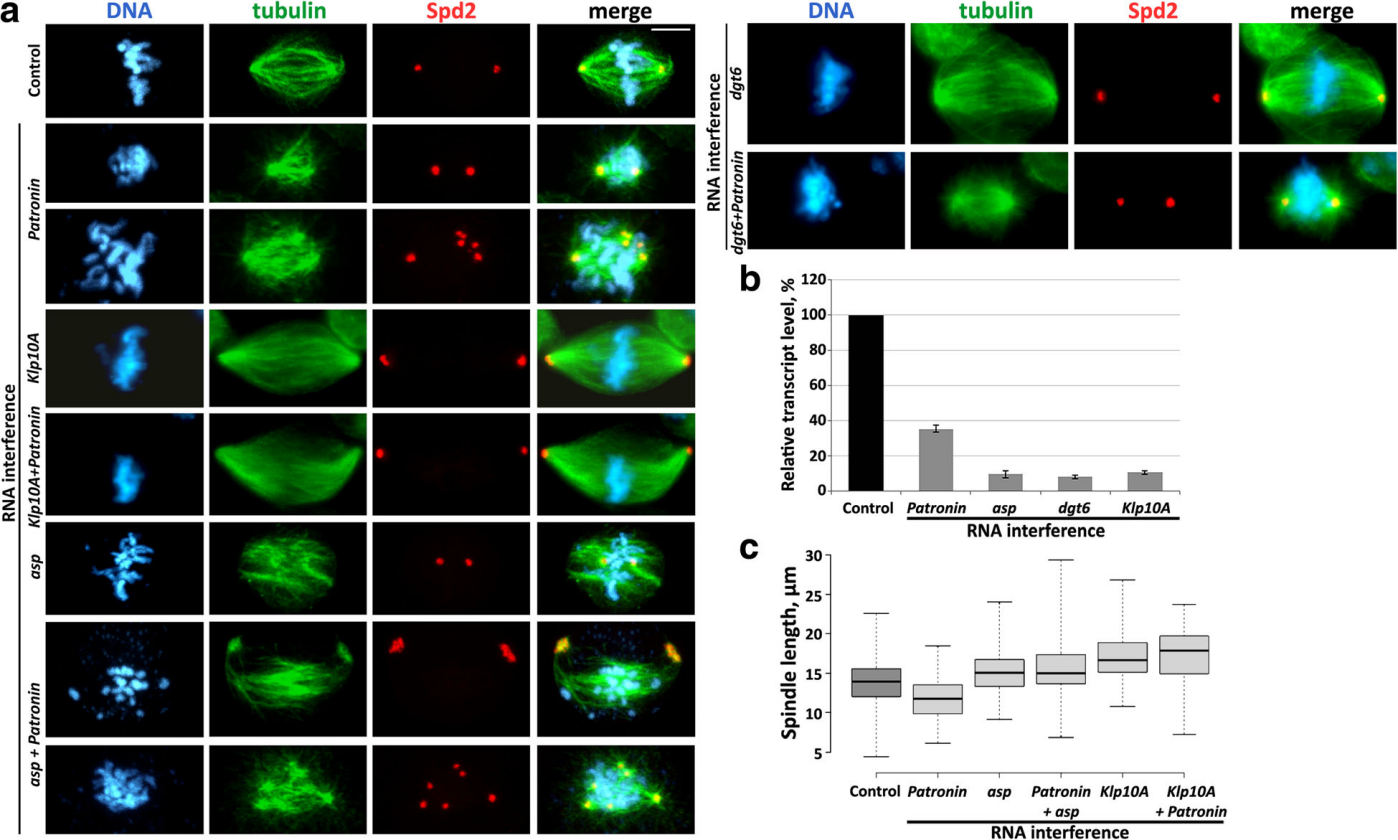
Mitotic phenotypes caused by co-depletion of Patronin and either Asp, Klp10A or Dgt6. (**a**) Examples of the mitotic phenotypes of cells depleted of Patronin, Klp10A, Asp, or Dgt6 and co-depleted of Patronin and Klp10A, Patronin and Asp, or Patronin and Dgt6. Cells were stained for DNA (DAPI, blue), α-tubulin (green) and the centrosomal marker Spd2 (red). Note the short collapsed spindles in Patronin-depleted cells and the long “rescued” spindles in cells co-depleted for either Patronin and Klp10A or Patronin and Asp. See text and Table 1 for detailed descriptions of the observed phenotypes. Scale bar, 5 μm. (**b**) RT-qPCR results showing that RNAi against *Patronin, asp, Klp10A,* or *dgt6* strongly reduces the level of the corresponding transcripts relative to a mock control that is set to 100%. For each gene, the reduction has been calculated by averaging the transcript levels detected in at least three independent RNAi experiments; *RpL32* was used as an endogenous reference gene. (**c**) Box and Whisker plots showing the quartile ranges of metaphase spindle length in control cells and cells depleted of Patronin, Asp, Klp10A, both Patronin and Asp, and both Patronin and Klp10A. Patronin-depleted cells have spindles significantly shorter than control spindles (*p* < 0.01), while Asp-depleted and Patronin and Asp co-depleted cells have spindles significantly longer than those of control cells (*p* < 0.01). Also note that Klp10A-depleted and Patronin and Klp10A co-depleted cells have spindles significantly longer than those of control cells (*p* < 0.01)

**Fig. 6:**
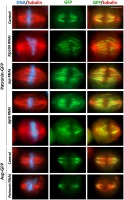
Localization of Patronin in cells depleted of proteins acting at MT minus ends (Klp10A, Asp, and Dgt6) and Asp localization in Patronin-depleted cells. Cells expressing Patronin-GFP or Asp-GFP were stained with anti-GFP antibodies (green) and anti-α-tubulin antibodies (red), and with DAPI to detect DNA (blue). Scale bar, 5 μm

Previous studies have shown that mitotic spindles of Patronin-depleted S2 cells are shorter than those of control cells and that this short-spindle phenotype is rescued by co-depletion of the MT depolymerase Klp10A [43]. However, these studies did not determine whether the rescued spindles have reacquired normal function. To address this question, we performed RNAi against *Patronin*, *Klp10A* and both *Patronin* and *Klp10A* and compared the mitotic phenotypes elicited by the three RNAi treatments. Patronin-depleted cells displayed many short spindles as previously described [43], with some of these spindles showing short and irregularly oriented MT bundles (Fig. 5a,c). In addition, Patronin depletion resulted in a significant increase in mitotic cells with multiple centrosomes (Fig. 5a; Table 1). However, despite the spindle defect, Patronin-depleted cells managed to divide. Bipolar cells with two centrosomes (one at each spindle pole) showed a frequency of anaphases slightly lower than that observed in control cells (Table 1), suggesting that in the absence of Patronin anaphase entry is delayed. Klp10A-depleted cells had very long spindles (Fig. 5a,c) and displayed many monastral spindles with two centrosomes at the center of the monaster, as previously described [23–25]; these cells showed frequencies of ana-telophases comparable to those observed in control cells, but higher frequencies of spindles with multiple centrosomes (Table 1). Consistent with previous results [43], in Patronin and Klp10A co-depleted cells, the short spindle phenotype was rescued, and spindles were even longer than control spindles (Fig. 5a,c). Also, the multiple-centrosome phenotype was rescued, as double RNAi cells displayed a normal frequency of cells with more than two centrosomes (Table 1). However, co-depleted cells showed a frequency of ana-telophases significantly lower than those observed in control cells and in cells depleted of Klp10A alone (Table 1). We also analyzed Patronin-GFP expressing cells exposed to RNAi against *Klp10A.* We found that *Klp10A* silencing does not substantially affect the association of Patronin with the spindle MT bundles (Fig. 6), and that the Patronin-stained MT bundles do not reach the centrosomes in prometaphase and metaphase cells. Collectively, these results indicate that although some of the spindle defects elicited by Patronin depletion are rescued by simultaneous loss of Klp10A, the “rescued spindles” are not fully functional and are defective in anaphase entry.

**Table 1:** Frequencies of mitotic figures observed after RNAi against the indicated genes. To determine the frequencies of cells with abnormal centrosome numbers, we examined all mitotic cells. The frequencies of the different mitotic figures were instead determined by examining only cells with two centrosomes. Thus, the total numbers of cells examined in the two types of analyses are different. Prometa, prometaphases; Meta, metaphases; Ana, anaphases; Telo, telophases; PMLES, prometaphase-like cells with elongated spindles

RNAi	No. of cells	No. of centrosomes		No. of cells	Prometa (%)	Meta (%)	Ana (%)	Telo (%)	PMLES (%)	Ana+Telo (%)
		1 cent. (%)	> 2 cent. (%)							
none (Control)	614	2.1	4.6	573	38.7	16.4	11.5	28.3	5.1	39.8
*Patronin*	430	5.6**	16.3**	336	37.5	17.9	8.6	28.9	7.1	37.5
*Klp10A*	235	4.3	9.4**	203	31.0	16.7	7.9	33.5	10.8**	41.4
*Patronin + Klp10A*	251	1.2	6.8	231	42.0	19.5	4.8**	23.4	10.4**	28.1**§
*asp*	273	5.9**	3.3	248	34.3	23.0	7.3	23.4	12.1**	30.6*
*Patronin + asp*	272	7.4**	22.4**	191	39.8	26.2	1.0**	11.5**	21.5**	12.6**§
*dgt6*	303	5.9**	4.0	273	45.4	19.0	2.6**	9.2**	23.8**	11.7**
*Patronin + dgt6*	260	2.3	13.1**	220	45.0	18.2	5.5*	19.5*	11.8**	25.0**

* and **, significantly different from control with *p* < 0.05 and < 0.01, respectively, in Chi-square test. §, significantly different from the appropriate “single” RNAi cells with *p* < 0.05, in Chi-square test

### Functional relationships between Patronin and Asp

To determine the functional relationships between Patronin and Asp, we asked whether the two proteins are mutually dependent for their localization on mitotic spindles. RNAi against *Patronin* in Asp-GFP expressing cells did not alter Asp localization at the spindle poles (Fig. 6). Similarly, RNAi-mediated depletion of Asp did not substantially affect Patronin association with the MT bundles of the spindle (Fig. 6). We next compared the mitotic phenotypes elicited by *asp* RNAi, *Patronin* RNAi and *asp* and *Patronin* double RNAi. As previously reported [16, 32, 33], we found that Asp-depleted cells exhibit abnormally long mitotic spindles often showing unfocused poles with detached centrosomes (Fig. 5a,c). Although recent work has shown that epithelial cells of *asp* mutants frequently exhibit supernumerary centrosomes [53], Asp depletion in S2 cells did not lead to an increase in the frequency of spindles associated with more than two centrosomes (Table 1). The latter finding is consistent with observations on brain cells from *asp* mutants [16], and suggests that the centrosomes of different cell types respond differently to Asp depletion. In *asp* and *Patronin* double RNAi cells, we found a combination of the phenotypes observed in cells depleted of either Patronin only or Asp only: unfocused spindles, spindles containing disorganized MT bundles, spindles with detached centrosomes, and an increase in the frequency of spindles associated with more than two centrosomes (Fig. 5a; Table 1). Cells depleted of both Patronin and Asp displayed abnormally long prometaphase/metaphase spindles, just as cells depleted of Asp only (Fig. 5c). We also found that the *asp* and *Patronin* double RNAi cells exhibit a frequency of PMLES (prometaphase-like cells with elongated spindles) that roughly corresponds to the sum of the PMLES frequencies observed in *asp* RNAi and *Patronin* RNAi cells (Table 1). PMLES (also called pseudo ana-telophases, PATs, [48]) are associated with chromosomes comprised of both sister chromatids and have been previously observed in cells with compromised kinetochore-MT interactions, such as those depleted of the centromere-specific histone H3 Cid/Cenp-A, the kinetochore components Ndc80, Nuf2 and Kmn1, or the kinetochore-associated MT depolymerase Klp67A [27, 48]. Finally, the *asp* and *Patronin* double RNAi cells showed a frequency of ana-telophases significantly lower than that observed in cells depleted of Patronin only or even Asp only (Table 1). This finding correlates with the relatively high frequency of PMLES observed in the *asp* and *Patronin* double RNAi cells and suggests that double RNAi cells are less able to satisfy the spindle assembly checkpoint (SAC) than cells exposed to RNAi against either *asp* or *Patronin* alone. In summary, both the protein localization results and the spindle morphology data suggest that Asp and Patronin play largely independent roles in S2 cell spindle assembly. However, loss of both proteins appears to have a synergistic effect in preventing anaphase entry, which might be related to a failure to satisfy the SAC.

### Functional relationships between Patronin and Dgt6

To determine the functional relationships between Patronin and the augmin complex, we focused on Dgt6, one of the best-characterized subunits of the complex [12, 31]. We performed RNAi against *dgt6* in Patronin-GFP expressing cells. This RNAi treatment did not grossly alter the Patronin localization pattern within the spindle. However, in several metaphase spindles the Patronin signals on the MT bundles were discontinuous, suggesting that augmin deficiency might slightly affect Patronin localization during mitosis (Fig. 6). We next examined the mitotic phenotype produced by double RNAi against *Patronin* and *dgt6* and compared it to that elicited by depletion of Patronin alone or Dgt6 alone. Consistent with previous results [12, 31], Dgt6-depleted cells showed spindles with a low MT density and a relatively high frequency of PMLES, suggesting defective MT-kinetochore interactions (Figs. 5a and 6; Table 1). *Patronin* and *dgt6* double RNAi cells showed a combination of the phenotypes observed in *Patronin* and *dgt6* RNAi cells (Fig. 5a; Table 1). Specifically, these double RNAi cells displayed several short spindles with a MT density lower than that seen in the short spindles of Patronin-depleted cells.

## Discussion

Previous studies have shown that Patronin binds the minus ends of interphase MTs, protecting them from depolymerization by Klp10A, and that Patronin depletion leads to short spindles, a phenotype that is rescued by Klp10A co-depletion. These results suggested that Patronin caps the minus ends of MTs, preventing their depolymerization by Klp10A [43]. Subsequent studies on the human homologues of Patronin, CAMSAP2 and CAMSAP3, showed that these proteins specifically bind the slowly growing MT minus ends, and remain associated with the MT lattice formed by minus end polymerization. Therefore, minus end growth results in the formation of extended stretches of CAMSAP-decorated MTs [37–39].

Our study has shown that Patronin binds bundled MTs within the mitotic spindles. In addition, analysis of fixed cells suggests that Patronin binds only a subset of the MT bundles within the spindle. This conclusion, however, should be taken with some caution. Given its dynamic behavior, it is indeed possible that Patronin binds MT bundles non-simultaneously and transiently, so that in fixed cells the MT bundles decorated by Patronin would be only those that were associated with Patronin at the moment of fixation. However, some regions of the spindle are never associated with Patronin, namely the middle of the central spindle where the plus ends of the anti-parallel MTs overlap and the region around the centrosome in prometaphase and metaphase cells; the latter region contains both the MT minus ends associated with the γ-TuRCs and the MT plus ends of the newly nucleated MTs. Thus, it appears that Patronin neither associates with regions that are particularly enriched in MT plus ends, nor with the γ-TuRC-capped minus ends embedded in the centrosome. However we observed some Patronin-stained MT bundles that appear to emanate from the centrosomes of prophase and telophase cells. We do not have an explanation for this finding, we can only suggest that in different mitotic phases the pericentrosomal regions contain different concentrations of free MT minus ends that can associate with Patronin.

Our analyses of the interactions among Patronin, Klp10A and Asp revealed that these proteins play largely independent roles in spindle assembly and function. Double RNAi cells displayed phenotypes that appear to be combinations of the phenotypes elicited by individual RNAi treatments. Specifically, it appears that both Klp10A depletion and Asp depletion have “dominant” effects in causing spindle elongation. Indeed, cells co-depleted of Patronin and Klp10A and cells depleted of Klp10A only exhibit spindles of similar length and not of an intermediate length between those of *Patronin* RNAi and *Klp10A* RNAi cells ([43], Fig. 5c). Similarly, Asp depletion has a dominant effect on spindle length in a Patronin-deficient background (Fig. 5c), as Asp-depleted cells exhibit spindles of the same length as those observed in Patronin and Asp co-depleted cells. Patronin and Dgt6 might also play independent roles during mitosis, although our observations raise the possibility that Dgt6 depletion affects Patronin association with metaphase MT bundles. However, a strong conclusion cannot be reached because augmin depletion results in spindles with reduced MT density and defective kinetochore fibers [12, 31], two conditions that could alter the pattern of Patronin localization along the spindle MTs.

The most interesting aspect of our study is the dynamic behavior of Patronin along the MT bundles. Our results indicate that in the MT bundles of prometaphase spindles, the Patronin front moves towards the spindle poles at a velocity of 1.44 ± 0.16 μm/min. This velocity is in the range of the MT flux velocity measured in S2 cells [25, 49]. The MT flux is the translocation of tubulin subunits toward the spindle poles generated by the addition of subunits to the MT plus ends at the kinetochore and the disassembly of the minus ends near the spindle poles [25]. What is then the role of Patronin in spindle assembly? We would like to propose a working hypothesis, which at the moment is rather speculative, but serves as a good starting point for further investigations. Current studies indicate that proteins of the CAMSAP family bind the growing minus ends of MTs. The prometaphase and metaphase MT bundles, which in S2 cells are mostly k-fibers composed of 11–15 MTs [54], could contain the minus ends of two kinds of MTs: MTs with their polymerizing plus ends embedded in the kinetochores, and MTs nucleated from the walls of preexisting MTs through an augmin-dependent mechanism. Interestingly, an electron microscopy-based study has shown that the minus ends of the latter category of MTs are often detached from their nucleation sites [55]. We propose that Patronin binds the minus ends of MTs growing from either the kinetochores or the lateral MT walls and helps them to adhere to the MT bundle exploiting the MT bundling activity of the CAMSAP proteins [35, 36]. Consistent with previous findings [37], we also envisage the intriguing possibility that the minus ends of both types of MTs can undergo a limited polymerization within the bundle. By accompanying the MT minus ends that are fluxing, and possibly polymerizing, towards the spindle poles, Patronin would coat and stabilize the prometaphase MT bundles, which would remain associated with Patronin during metaphase and early anaphase.

A similar model could be extended to the central spindle bundles, which are enriched in augmin in both *Drosophila* and human cells [31, 56]. There is evidence that the central spindle assembles from MTs nucleated by the centrosomes, by (or near) the chromosomes and via the augmin pathway ([56] and references therein). We propose that Patronin binds the minus ends of the augmin-dependent MTs, and possibly other MT minus ends, contributing to the stabilization of the bundles formed by these MTs during central spindle assembly.

A model for prometaphase and metaphase MT bundle formation has been recently proposed by Ito and Goshima [33]. They suggested that the minus ends of the intraspindle MTs generated via the augmin pathway associate with Asp, which would help to cross-link them with the long “mother” MTs. They further proposed that Asp assists in linking the new, augmin-dependent MTs to the MT bundles during their poleward movement. This model does not conflict with our Patronin-based hypothesis, and we instead believe that the two models are mutually compatible. Verifying our model, defining the precise relationships between Patronin and Asp in assisting intraspindle MT formation and behavior, and understanding whether a limited minus end polymerization occurs within the kinetochore MT bundles are very interesting topics to be addressed in future research.

## Conclusions

Previous work has shown that Patronin and its human homologues bind the minus ends of interphase MTs. The work described here indicates that Patronin does not bind the minus ends of the spindle MTs capped by the γ-TuRCs. Our results led us to propose that Patronin binds the free minus ends of MTs generated from the walls of preexisting MTs via the augmin pathway, contributing to MT bundle formation. However, this is only a working hypothesis that has to be tested in future experiments.

## Additional files

Additional file 1: **Figure S1.** Additional examples of Patronin-GFP localization in mitotic cells. S2 cells expressing Patronin-GFP and Cherry-tubulin were fixed and stained with anti-GFP (green) and anti-α-tubulin (red) antibodies, and with DAPI to detect DNA (blue). Prometa, prometaphase; Meta, metaphase; Telo, telophase. Note that only a subset of the MT bundles associate with Patronin-GFP. The white scale bar (5 μm) refers to all cells except telophase (red scale bar, 4 μm). (TIF 2736 kb)

Additional file 2: **Figure S2.** An additional example of the dynamic behavior of Patronin-GFP during prometaphase. Stills from a time-lapse video of an S2 cell prometaphase expressing Patronin-GFP (green) and Cherry-tubulin (red). The numbers at the top of each frame indicate the time (min:sec) elapsed from the beginning of imaging. Note the dynamic behavior of Patronin. Scale bar, 5 μm. (TIF 742 kb)

Additional file 4: **Figure S3.** An additional example of Patronin-GFP behavior during meta-telophase. Stills from a time-lapse video of an S2 cell expressing Patronin isoform A-GFP (green) and Cherry-tubulin (red), followed from metaphase until telophase. The numbers at the top of each frame indicate the time (min:sec) elapsed from the beginning of imaging. The chromosomes appear as dark spots in the green Patronin isoform A-GFP background. Scale bar, 5 μm. (TIF 2780 kb)

## Abbreviations

+TIP: Microtubule plus end-tracking protein
BSA: Bovine serum albumin
CAMSAP: Calmodulin-regulated spectrin-associated protein
cDNA: Complementary DNA
CDS: Coding DNA sequence
DAPI: 4′,6-diamidino-2-phenylindole
FITC: Fluorescein isothiocyanate
GFP: Green fluorescent protein
k-fiber: Kinetochore fiber
MT: Microtubule
PBS: Phosphate-buffered saline
PCR: Polymerase chain reaction
PMLES: Prometaphase-like cells with elongated spindles
qPCR: Quantitative PCR
RNAi: RNA interference
RT-qPCR: Reverse transcription followed by quantitative PCR
−TIP: Microtubule minus end-tracking protein
γ-TuRC: γ-tubulin ring complex
