# Publisher Correction: The role of Patronin in *Drosophila* mitosis

**DOI:** 10.1186/s12860-019-0196-1

**Published:** 2019-07-08

**Authors:** Gera A. Pavlova, Alyona V. Razuvaeva, Julia V. Popova, Evgeniya N. Andreyeva, Lyubov A. Yarinich, Mikhail O. Lebedev, Claudia Pellacani, Silvia Bonaccorsi, Maria Patrizia Somma, Maurizio Gatti, Alexey V. Pindyurin

**Affiliations:** 1 Institute of Molecular and Cellular Biology, Siberian Branch of the Russian Academy of Sciences, Novosibirsk, 630090 Russia; 2 Novosibirsk State University, Novosibirsk, 630090 Russia; 3 IBPM CNR and Department of Biology and Biotechnology, Sapienza University of Rome, 00185 Rome, Italy

**Correction to: BMC Mol Cell Biol (2019) 20(Suppl 1):7**

**https://doi.org/10.1186/s12860-019-0189-0**

During production of the original article [1], there was a technical error that resulted in author corrections not being rendered in the PDF version of the article.

This error has now been corrected in the PDF of the published article.

For the full details of the corrections that were previously not rendered in the PDF, please see Additional file 1.

The publisher apologizes for this error.

## Additional file

Additional file 1: Please review for the full details of the corrections that were previously omitted in the PDF. (PDF 149 kb)
