# Supplementary figures and images for: The role of Patronin in Drosophila mitosis
Source: BMC Mol Cell Biol. 2019 Apr 17;20(Suppl 1):7. doi: 10.1186/s12860-019-0189-0 (PMC6469034; doi:10.1186/s12860-019-0189-0)

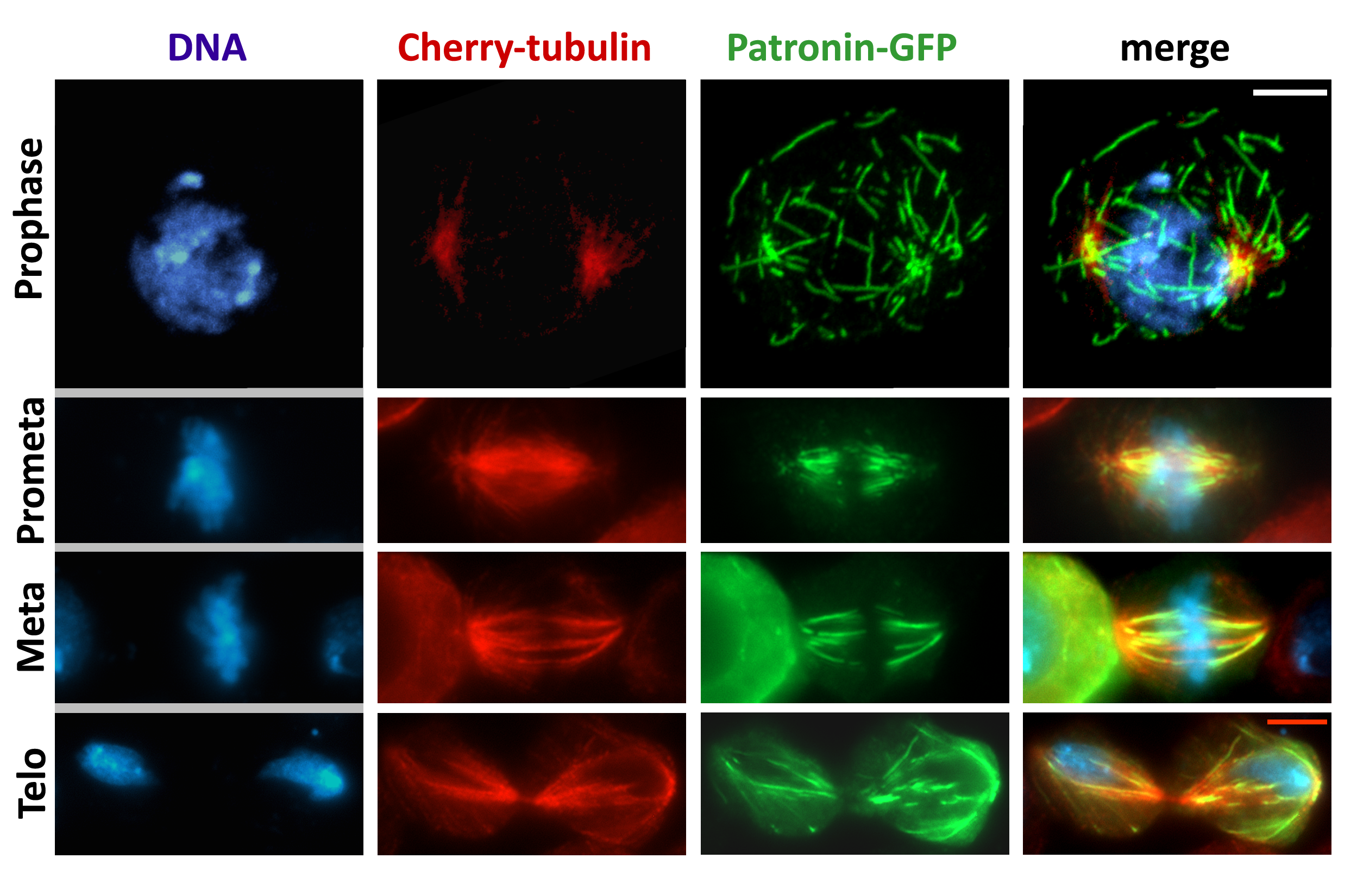

Supplement: Supplementary file 3 — Figure S1. Additional examples of Patronin-GFP localization in mitotic cells. S2 cells expressing Patronin-GFP and Cherry-tubulin were fixed and stained with anti-GFP (green) and anti-α-tubulin (red) antibodies, and with DAPI to detect DNA (blue). Prometa, prometaphase; Meta, metaphase; Telo, telophase. Note that only a subset of the MT bundles associate with Patronin-GFP. The white scale bar (5 μm) refers to all cells except telophase (red scale bar, 4 μm). (TIF 2736 kb) [file 12860_2019_189_MOESM1_ESM.tif]

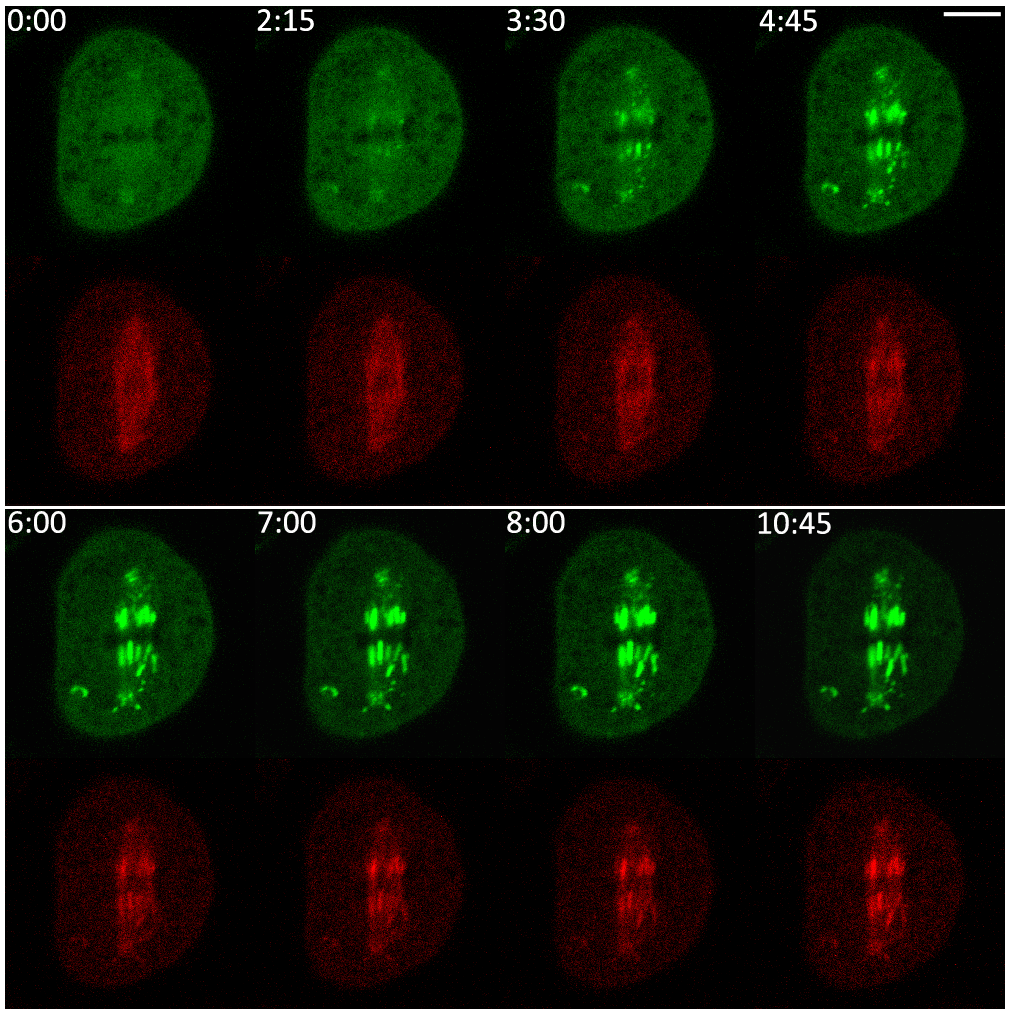

Supplement: Supplementary file 4 — Figure S2. An additional example of the dynamic behavior of Patronin-GFP during prometaphase. Stills from a time-lapse video of an S2 cell prometaphase expressing Patronin-GFP (green) and Cherry-tubulin (red). The numbers at the top of each frame indicate the time (min:sec) elapsed from the beginning of imaging. Note the dynamic behavior of Patronin. Scale bar, 5 μm. (TIF 742 kb) [file 12860_2019_189_MOESM2_ESM.tif]

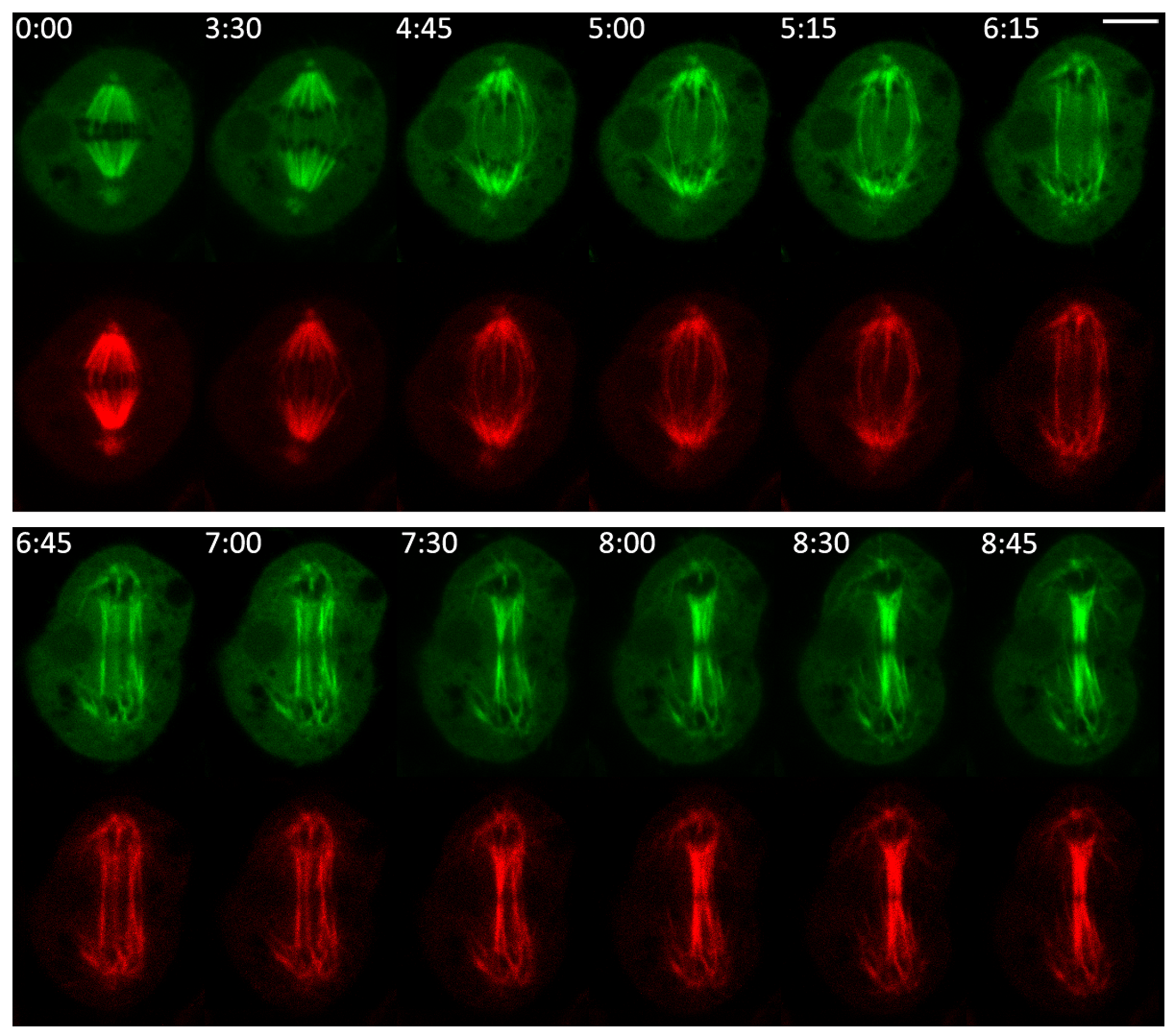

Supplement: Supplementary file 5 — Figure S3. An additional example of Patronin-GFP behavior during meta-telophase. Stills from a time-lapse video of an S2 cell expressing Patronin isoform A-GFP (green) and Cherry-tubulin (red), followed from metaphase until telophase. The numbers at the top of each frame indicate the time (min:sec) elapsed from the beginning of imaging. The chromosomes appear as dark spots in the green Patronin isoform A-GFP background. Scale bar, 5 μm. (TIF 2780 kb) [file 12860_2019_189_MOESM4_ESM.tif]
