# Supplementary material for: Publisher Correction: The role of Patronin in Drosophila mitosis
Source: BMC Mol Cell Biol. 2019 Jul 8;20:24. doi: 10.1186/s12860-019-0196-1 (PMC6615211; doi:10.1186/s12860-019-0196-1)
Supplement: Supplementary file 1 — Please review for the full details of the corrections that were previously omitted in the PDF. (PDF 149 kb) [file 12860_2019_196_MOESM1_ESM.pdf]

Section	Paragraph	Correction
Author list		"Alena" replaced by "Alyona"
Affiliation 1		"[1]Department of Regulation of Genetic Processes, Institute of Molecular and Cellular Biology, Siberian Branch of RAS, Novosibirsk 630090, Russia"<br><br>replaced by<br><br>"[1]Institute of Molecular and Cellular Biology, Siberian Branch of the Russian Academy of Sciences, Novosibirsk 630090, Russia"
Affiliation 2		"[2]Laboratory of Structural, Functional and Comparative Genomics, Novosibirsk State University, Novosibirsk 630090, Russia"<br><br>replaced by<br><br>"[2]Novosibirsk State University, Novosibirsk 630090, Russia"
Abstract Background		"on" in "Previous work has shown that Patronin stabilizes the minus ends on non-mitotic MTs" replaced by "of"
Abstract Results		Comma removed in "Here we have explored the role of Patronin in *Drosophila* mitosis, using S2 tissue culture cells"
Abstract Conclusion		"wall" in "including those generated from the wall of preexisting MTs via" replaced by "walls"
Background	2nd	"+ TIP" in "while Klp10A behaves as a + TIP factor in interphase" replaced by "+TIP"
Background	2nd	"+TIPs include the End Binding proteins (e.g., EB1 and EB3 in vertebrates; DmEB1 in *Drosophila*) that accumulate at MT plus ends where they recruit other +TIPs MT polymerases such as XMAP215/ch-TOG (Mini spindles, or Msps, in *Drosophila*); "<br><br>replaced by<br><br>"+TIPs include the End Binding proteins (e.g., EB1 and EB3 in vertebrates; DmEB1 in *Drosophila*) that accumulate at MT plus ends where they recruit other proteins such as the XMAP215/ch-TOG MT polymerase (Mini spindles, or Msps, in *Drosophila*), "
Background	3rd	"-TIP" replaced by "−TIP"
Background	3rd	"ends" in "in the few cases when MT minus ends polymerization was detected" replaced by "end"
Background	4th	Two instances of "-TIP" replaced by "−TIP"

Background	5th	"CAMSAP2 and CAMSAP3 function in interphase but are removed from the spindle MTs due to phosphorylation [38, 41]."<br><br>replaced by<br><br>"CAMSAP2 and CAMSAP3 function in interphase but fail to associate with prometaphase and metaphase spindles due to phosphorylation [38, 41]. "
Background	6th	"*ssp4/Patronin*" replaced by "*Patronin/ssp4*"
Background	6th	"Kp10A" replaced by "Klp10A"
RNAi	1st	"cells" replaced by "cell"
	2nd	Inserted "Genomic DNA was eliminated using the RapidOut DNA Removal Kit (Thermo Fisher Scientific)." after "Total RNA was isolated…instructions."
	2nd	Removed "Genomic DNA was eliminated using the RapidOut DNA Removal Kit (Thermo Fisher Scientific)." after "……..Fisher Scientific)."
	2nd	"http://biolabmix.ru/" replaced by "http://biolabmix.ru/en/"
Generation of stable S2 cell lines expressing fluorescently-tagged proteins	1st	"synthesized" replaced by "made"
	1st	Inserted "isolated" after "…….from total RNA"
	1st	Removed "[1]" after "*Sb*"
	3rd	"All these" replaced by "These"
	3rd	"metallothionein A (MtnA)" replaced by "*Metallothionein A* (*MtnA*)"
Cytological procedures	1st	"antibodies (1:100, [51])" replaced by "(1:1000, [51])"
	1st	"100x/1.30" replaced by "100×/1.30"
Figure 1 legend		"anti-tubulin" replaced by "anti-α-tubulin"
Figure 1 legend		Inserted "Prometa, prometaphase; Meta, metaphase; Ana, anaphase; Telo, telophase." after "……. DAPI to detect DNA (blue)."
Patronin behavior in mitotic spindles is dynamic	1st	removed split in "Patronin-GFP"
Functional relationships between Patronin and Klp10A	1st	Table 1 was linked
Figure 5 caption		Inserted "Mitotic phenotypes caused by co-depletion of Patronin and either Asp, Klp10A or Dgt6. (a)" before "Examples of the mitotic….."
Figure 5 caption		"…………least three independent RNAi experiments."<br><br>replaced by<br><br>"……least three independent RNAi experiments; *RpL32* was used as an endogenous reference gene."
Table 1 caption		Two instances of "cell" replaced by "cells"
Table 1 caption		"Prometaphase-like" replaced by "prometaphase-like"

Table 1 legend		Two instances of "Chi square" replaced by "Chi-square"
Figure 6 caption		"cell" replaced by "cells"
Functional relationships between Patronin and Asp	heading	"asp" replaced by "Asp"
	1st	"This latter" replaced by "The latter"
	1st	"cells" replaced by "cell"
	1st	"we observed" replaced by "we found"
	1st	"(ProMetaphase-Like cells with Elongated Spindles)"<br><br>replaced by<br><br>"(prometaphase-like cells with elongated spindles)"
	1st	"Pseudo Ana-Telophases" replaced by "pseudo ana-telophases"
Discussion	2nd	"MT overlap" replaced by "MTs overlap"
	2nd	"these regions contain" replaced by "the latter region contains"
	2nd	"does not" replaced by "neither"
	2nd	"associate" replaced by "associates"
	2nd	"Although" replaced by "However"
	2nd	"cells, we" replaced by "cells. We"
	2nd	"find-ing. We" replaced by "finding, we"
	2nd	"bind" replaced by "associate with"
	4th	"bundle, exploiting" replaced by "bundle exploiting"
	4th	"spindles" replaced by "spindle"
	6th	"Asp that helps" replaced by "Asp, which would help"
Acknowledgements	1st	"SB RAS" replaced by "of the Siberian Branch of the Russian Academy of Sciences"
	2nd	"18–34-00688" replaced by "18-34-00688"
	2nd	"0310–2018-0009 to AVP" replaced by "0310-2019-0005"
	2nd	"18–34-00688" replaced by "18-34-00688"
Conclusions		"wall" in "Patronin binds the free minus ends of MTs generated from the wall of preexisting MTs via" replaced by "walls"
Abbreviations		"-TIP" replaced by "–TIP"
Additional file 1 caption		Inserted "Prometa, prometaphase; Meta, metaphase; Telo, telophase." before "Note that only a……"
Additional file 3 caption		Deleted "and Cherry-tubulin (red)"
Additional file 5 caption		"metaphase" replaced by "anaphase"
References	2	"Reconstituition" replaced by "Reconstitution"
	16	"asp" replaced by "Asp"
	23	"orbit" replaced by "Orbit"
	28	"Centre" replaced by "centre"
	32	"dynein" replaced by "Dynein"
	32	"(asp)" replaced by "(Asp)"
	33	"asp" replaced by "Asp"

	36	“Proc Natl Acad Sci” replaced by “Proc Natl Acad Sci USA”
	37	“Proc Natl Acad Sci” replaced by “Proc Natl Acad Sci USA”
	45	“Patronin/shot” replaced by “Patronin/Shot”
	53	“dynein” replaced by “Dynein”
	53	“mud” replaced by “Mud”
	53	“asp” replaced by “Asp”
